# Nocardia Subretinal Abscess: A Rare and Challenging Case Report

**DOI:** 10.1155/2021/8876864

**Published:** 2021-01-09

**Authors:** Ana Maria Cunha, Marta Silva, Ana Catarina Pedrosa, Fernando Falcão-Reis, Luís Figueira

**Affiliations:** ^1^Department of Ophthalmology, Centro Hospitalar Universitário de São João, Porto, Portugal; ^2^Department of Surgery and Physiology, Faculty of Medicine, University of Porto, Porto, Portugal; ^3^Department of Pharmacology and Therapeutics, Faculty of Medicine of the University of Porto, Porto, Portugal; ^4^Center for Drug Discovery and Innovative Medicines (MedInUP), University of Porto, Porto, Portugal

## Abstract

**Purpose:**

To describe a positive clinical response of a patient with submacular *Nocardia* abscess due to a rapid and efficient treatment. *Case report*. We describe a case of a 50-year-old man with a painless visual decline of the left eye. Four years later, he had been diagnosed with systemic nocardiosis. Examination of the left eye revealed a submacular white mass with fluffy borders and another smaller white lesion, with well-defined borders, in the inferior temporal vascular arch. A systemic antibiotic treatment with SMX-TMP and intravenous imipenem and a single intravitreal injection of bevacizumab was performed.

**Conclusion:**

Prompt diagnosis and treatment ensured an expeditious resolution of the abscess and significant improvement of visual acuity. The diagnostic approach of a high index of suspicion coupled with directed treatment is required when dealing with subretinal inflammatory lesions.

## 1. Introduction

Nocardia is an aerobic, gram-positive, Actinomycetales bacteria, which is ubiquitous in soil [1]. It usually manifests as an opportunistic infection in immunocompromised individuals [2].

Typically, systemic involvement includes the lungs, brain, kidneys, skin, and joints [3]. Initial clinical manifestations comprise mainly of pulmonary symptoms. Hematogenous spread to other organs is observed in approximately one-third of patients [4].

The ocular manifestations can be classified by mechanism of inoculation as exogenous, when occurs posttraumatically and endogenous, secondary to hematogenous spread [5, 6].

Ocular infection can manifest in various forms including keratitis, conjunctivitis, scleritis, periorbital infections, subretinal or choroidal abscess, and endophthalmitis [4, 7–9]. Only a few reports have posterior segment involvement. Choroidal and retina involvement mostly leads to loss of vision and, in some cases, to evisceration or enucleation [8, 10].

We describe a positive clinical response of a patient with submacular Nocardia abscess due to a rapid and efficient treatment.

## 2. Case Report

We describe a case of a 50-year-old man with a 3-day history of painless visual decline in the left eye. His past medical history included a diagnosis of pulmonary silicosis with progressive pulmonary fibrosis. In February 2016, he had been diagnosed with systemic nocardiosis with lung, cerebral, and skin abscess and had received sulfamethoxazole-trimethoprim (SMX-TMP) oral monotherapy until December 2017.

**Figure 1:**
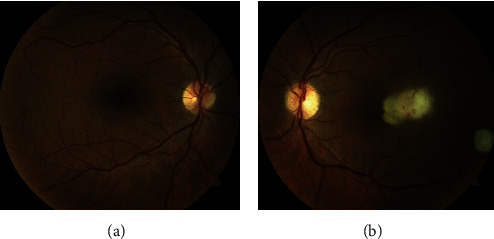
(a) Normal fundus-photography of the right eye. (b) Fundus-photography of the left eye picturing a whitish, fluffy subretinal macular mass and another smaller white lesion, with well-defined limits, in the inferior temporal vascular arch.

**Figure 2:**
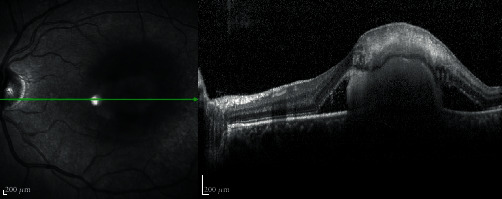
SD-OCT scan of the subretinal mass with disorganization of retinal structures with subretinal fluid.

**Figure 3:**
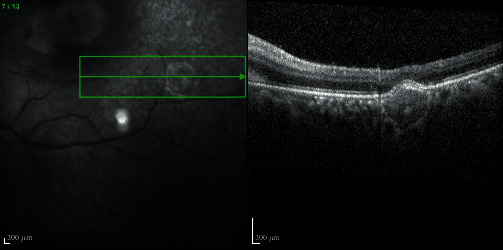
SD-OCT scan of the lesion in the inferior temporal vascular arch showing a well define mass in the choroidal space.

**Figure 4:**
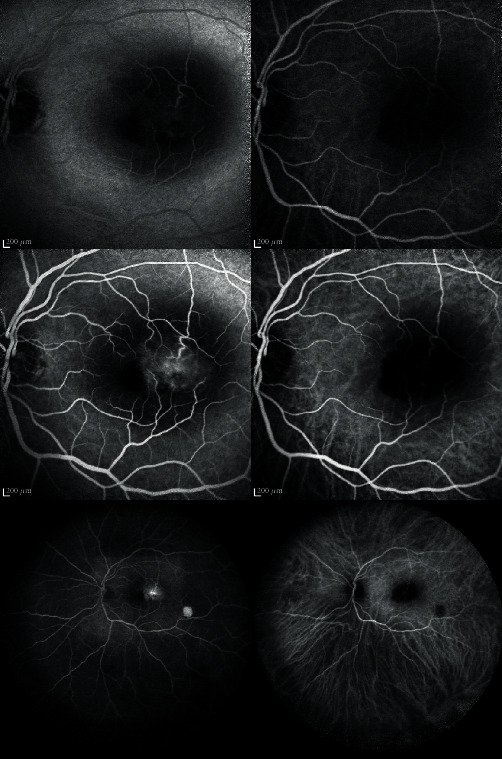
FA and ICG in the initial presentation at 0 : 56 min, 2 : 41 min, 4 : 01 min.

In October 2019, he presented, in the ophthalmic emergency room, with a visual acuity of 10/10 in the right eye and 4/10 in the left eye. The anterior examination in both eyes was unremarkable and intraocular pressure normal. Posterior segment examination of the right eye was normal (Figure 1(a)), and the left eye revealed absence of vitritis and a one disk diameter submacular white mass with fluffy borders and some intraretinal hemorrhages and another smaller white lesion, with well-defined borders, in the inferior temporal vascular arch (Figure 1(b)). The SD-OCT of the main macular lesion showed a subretinal mass with disorganization of retinal structures in association with subretinal fluid (Figure 2). The SD-OCT scan of the smaller white lesion in the inferior temporal vascular arch revealed a well define mass in the choroidal space (Figure 3). Fluorescein angiography (FA) revealed an area of early hypofluorescence, which progressed to a hyperfluorescence lesion with fluorescein leakage in late stages and a hyperfluorescence of the smaller lesion in the inferior temporal vascular arch without diffusion. Indocyanine Green Angiography (ICG) revealed a hypofluorescence in all stages (Figure 4).

Due to clinical chorioretinal lesion presentation, medical record, and additional symptoms (fever and dry cough), the patient began empiric systemic antibiotic treatment with SMX-TMP and intravenous imipenem.

**Figure 5:**
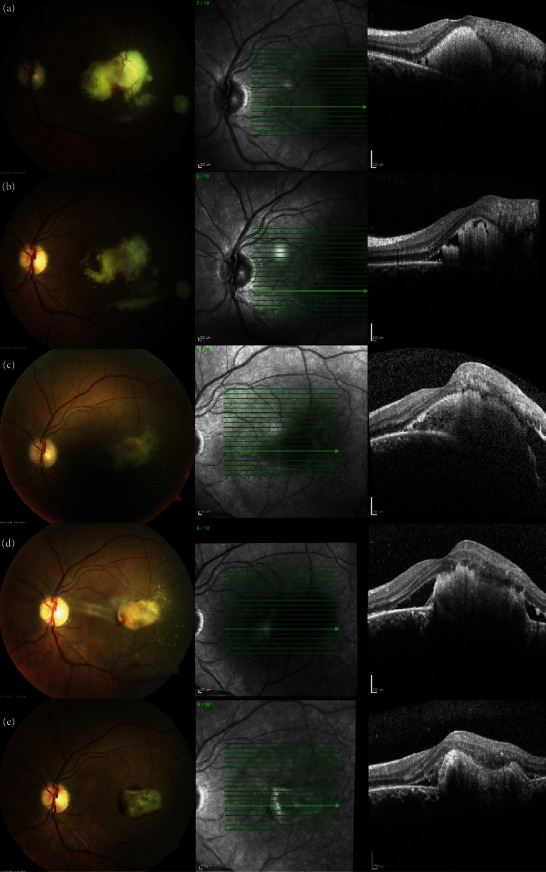
Chronological evolution of the macula lesion during treatment. (a) Macular lesion enlargement with neurosensorial detachment and a pseudohypopyon aspect at D3 of treatment. (b–e) Macular lesion regression and decreased subretinal fluid.

After 3 days, the left eye visual acuity decreased to counting fingers. Although, in the ophthalmological exam of the anterior examination remains unremarkable and without vitritis in the posterior segment, the macular lesion enlarges had more intraretinal hemorrhages, neurosensorial detachment, and a pseudohypopyon aspect (Figures 5(a) and 5(b)).

Initial systemic study presented with an increased C-Reactive Protein (CRP) (84.8), but unremarkable virologic study (HIV, HCV, toxoplasmosis, *Treponema pallidum*, CMV, EBV, VHS1, and VHS2 negative). Further systemic workup with cranial MRI and thoraco abdominopelvic CT scan revealed no signs of active infection. Although the anterior chamber tap for aqueous humour CRP microbiological analysis was negative for *Mycobacterium tuberculosis*, Herpes virus, *Toxoplasma*, Syphilis, and *Nocardia*, the analysis of the bronchial secretion's specimen identified *Nocardia abscessus*.

After 6 days of treatment, the macular lesion began to decrease in size and decrease the subretinal fluid (Figures 5(c)–5(e)). The patient was treated with TMP-SMX and intravenous imipenem for three weeks, and later, with oral TMP-SMX and cefixime.

**Figure 6:**
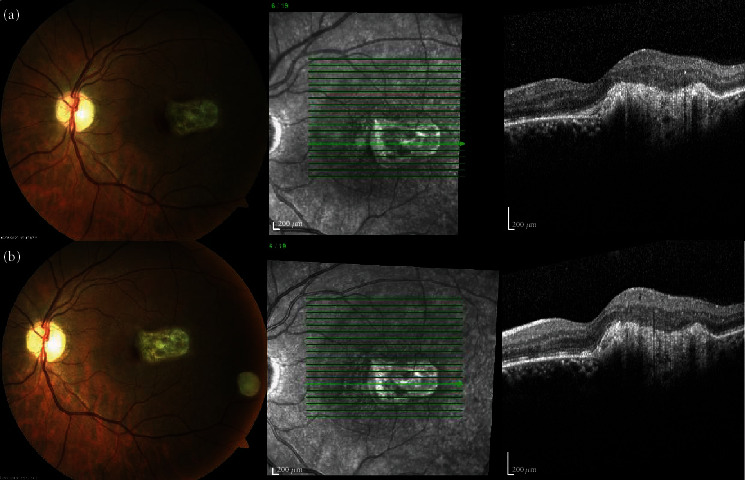
Chronological evolution of the macula lesion during treatment. One months (a) and two months (b) after the intravitreal injection, fibrotic scarring lesion without subretinal and intraretinal fluid and decreased intraretinal hemorrhages were identified.

**Figure 7:**
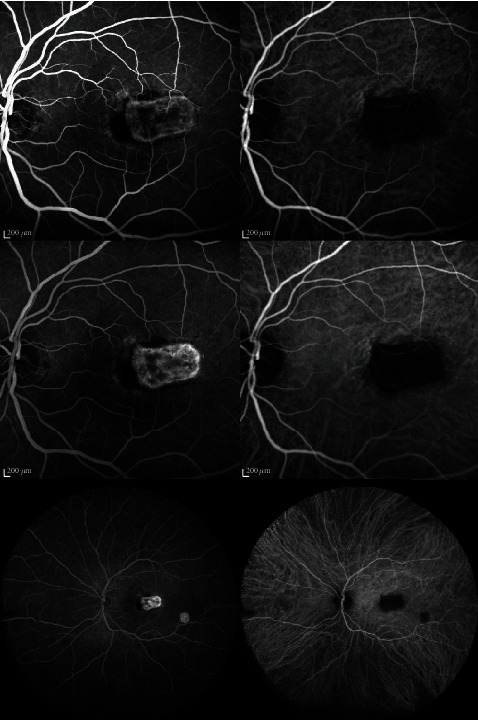
FA revealed a hyperfluorescence staining in macular and peripheric lesion. ICG revealed a hypofluorescence in all stages. (0 : 52 min; 2 : 16 min; 3 : 36 min).

He was discharged after 44 days of treatment. At this time, the left eye visual acuity was 0.008, and the macular lesion was smaller, but intraretinal hemorrhages had increased, and it kept intraretinal and subretinal fluid (Figure 5(e)). For this reason, it was performed one intravitreal injection of bevacizumab 1.25 mg. One month after the intravitreal injection, while continuing systemic treatment with oral TMP-SMX and cefixime, the ophthalmic examination revealed visual acuity of 10/10 in the right eye and 5/10 in the left eye. Slit lamp examination was quiet and intraocular pressure normal. Posterior segment exam revealed subretinal fibrosis in macula. The SD-OCT revealed fibrotic scarring lesion without subretinal and intraretinal fluid (Figures 6(a) and 6(b)). FA revealed an hyperfluorescence staining of the macular and peripheric lesions, without signs of activity. ICG revealed a hypofluorescence in all stages (Figure 7).

The patient will continue systemic treatment with oral TMP-SMX and cefixime until perform 12 months.

## 3. Discussion

Ocular nocardiosis usually presents with insidious and painless vision loss [11]. Most commonly, it appears in association with immunodeficiency causes such as AIDS or pharmacological immunosuppression in cases of stem cell transplantation [12], organ transplant [13], or malignancy [14]. Nevertheless, a few cases of the infection have also been reported in immunocompetent patients [15].

When ocular manifestations were present, 70% had pulmonary disease and 50% had central nervous system diseases [11]. For this reason, a pulmonary examination is warranted in all cases of *Nocardia* choroidal abscesses, with further evaluation of other organ systems guided by individual patient symptoms [8]. In our case, we identified pulmonary and ocular manifestations of *Nocardia*.

Severity of the ocular manifestations can be highly variable, which makes diagnosis difficult. Ocular *Nocardia* presentation can range from minimal inflammation to significant vitritis [16]. The fundoscopic findings are usually subretinal abscess, chorioretinal exudate, or inflammatory mass with associated superficial or intraretinal hemorrhages. In some cases, it is reported retinal detachment [8, 11]. Scleral thickening, choroidal edema, and panophthalmitis have been also described [10]. MRI may be helpful for quantifying the ocular involvement [11].

Consonant to our case, the unilateral and poor visual acuity at presentation and the location at the posterior pole are common [8]. The predisposition for the posterior pole may be associated with the higher choroidal blood flow in this region [8, 17].

The early diagnostic and specific treatment of *Nocardia* can be difficult by the unspecific manifestations and the broad differential diagnosis encompassing viral retinitis, toxoplasmosis, fungal endophthalmitis, and neoplasia [11]. In previous reported clinical cases, diagnosis delay could be 2 to 6 weeks, which can lead to a pathogen proliferates rapidly [8]. Viral retinitis generally can be distinguished from *Nocardia* choroidal abscesses as the former develops retinal necrosis accompanied by anterior and/or intermediate uveitis (i.e., in acute retinal necrosis) [8]. Ocular toxoplasmosis usually manifests as retinal necrosis, vitritis, inactive scars, and vasculitis [11, 18]. Fungal infections can have different presentations, like *Candida* endophthalmitis which usually presents as a small cream-coloured peripheral choroid lesion and flocculent fungal balls in vitreous [19] or endogenous *Aspergillus* endophthalmitis that has a more rapid progression and larger areas of infarcted haemorrhagic chorioretinitis [20]. Only a few other bacterial species are mentioned in literature, such as *Pseudomonas aeruginosa*, *Streptococcus viridans*, or *Klebsiella pneumoniae*, that cause subretinal abscess formation [11]. Neoplastic diseases, mainly choroidal metastases, are usually multifocal and occur in patients with a history of malignancy [21].

Therefore, the clinical presentation of a subretinal white fluffy abscess, which can progress to pseudohypopyon aspect, the superficial or intraretinal hemorrhages and the absence of vitritis in a patient with pulmonary disease or medical record of pulmonary disease, should lead to suspicion of nocardial aetiology. In the case of our patient, pulmonary nocardiosis had indeed been diagnosed in the past, rendering this specific diagnosis more likely.

Some reported cases have been treated with intravitreal injection of amikacin, due to the antibiotic sensitivity profile, but with minimal guidance on the frequency of injections [8]. However, macular ischemia following intravitreal amikacin is a serious risk to consider with individualized discussions [22]. In our case, due to the good response to systemic treatment and the aforementioned risk of macular ischemia, we decided to postpone intravitreal injection. Intravitreal injections of bevacizumab have been used to treat neovascular membranes or macular edema. In this case, the lesion had exudation, intraretinal and subretinal fluid caused by vascular component. Intravitreal injection of bevacizumab was cooperative in the regression of the vascular component and the resolution of the fluid.

## 4. Conclusion

The visual prognosis is variable and depends on the location of the abscess, as well as the tissue destruction, which limits establishing prognosis. Our patient had severely reduced vision in the setting of a large submacular abscess. Nevertheless, a quick diagnosis hypothesis and prompt systemic treatment ensured a marked resolution of the abscess and significant improvement of visual acuity. This case highlights that a diagnostic approach and a high index of suspicion (even if sometimes purely clinical one) coupled with directed treatment is required when dealing with subretinal inflammatory lesions.

## Data Availability

Data supporting our study can be provided upon request.
